# Use of Patient-Specific 3D Models in Paediatric Surgery: Effect on Communication and Surgical Management

**DOI:** 10.3390/jimaging12020056

**Published:** 2026-01-26

**Authors:** Cécile O. Muller, Lydia Helbling, Theodoros Xydias, Jeanette Greiner, Valérie Oesch, Henrik Köhler, Tim Ohletz, Jatta Berberat

**Affiliations:** 1 Paediatric Surgery Department, Cantonal Hospital Aarau, Tellstrasse 25, 5001 Aarau, Switzerland; lydia.helbling@ksa.ch (L.H.); valerie.oesch@ksa.ch (V.O.); 2 Paediatric Radiology Department, Cantonal Hospital Aarau, Tellstrasse 25, 5001 Aarau, Switzerland; theodoros.xydias@ksa.ch; 3 Paediatric Oncology Department, Cantonal Hospital Aarau, Tellstrasse 25, 5001 Aarau, Switzerland; jeanette.greiner@ksa.ch; 4 Paediatric Department, Cantonal Hospital Aarau, Tellstrasse 25, 5001 Aarau, Switzerland; henrik.koehler@ksa.ch; 5 Radiology Department and 3D Lab, Cantonal Hospital Aarau, Tellstrasse 25, 5001 Aarau, Switzerland; tim.ohletz@ksa.ch; 6 Neuroradiology Department, Cantonal Hospital Aarau, Tellstrasse 25, 5001 Aarau, Switzerland; jatta.berberat@ksa.ch

**Keywords:** patient-specific 3D models, paediatric rare diseases, communication

## Abstract

Children with rare tumours and malformations may benefit from innovative imaging, including patient-specific 3D models that can enhance communication and surgical planning. The primary aim was to evaluate the impact of patient-specific 3D models on communication with families. The secondary aims were to assess their influence on medical management and to establish an efficient post-processing workflow. From 2021 to 2024, we prospectively included patients aged 3 months to 18 years with rare tumours or malformations. Families completed questionnaires before and after the presentation of a 3D model generated from MRI sequences, including peripheral nerve tractography. Treating physicians completed a separate questionnaire before surgical planning. Analyses were performed in R. Among 21 patients, diagnoses included 11 tumours, 8 malformations, 1 trauma, and 1 pancreatic pseudo-cyst. Likert scale responses showed improved family understanding after viewing the 3D model (mean score 3.94 to 4.67) and a high overall evaluation (mean 4.61). Physicians also rated the models positively. An efficient image post-processing workflow was defined. Although manual 3D reconstruction remains time-consuming, these preliminary results show that colourful, patient-specific 3D models substantially improve family communication and support clinical decision-making. They also highlight the need for supporting the development of MRI-based automated segmentation softwares using deep neural networks, which are clinically approved and usable in routine practice.

## 1. Introduction

Paediatric patients with rare diseases such as malformations or tumours require long-term follow-up with their surgical teams. Maintaining trust and effective communication with their families is essential. In this context, advanced tools such as patient-specific 3D models—part of the emerging “digital twin” concept [1]—are proving valuable. These models are now well known to enhance anatomical understanding, improve physician–parent communication, and support medical education and surgical planning.

While the use of 3D modelling is well established in adult surgical fields—including orthopaedics, neurosurgery, abdominal, cardiac, and head and neck surgery—its application in paediatrics remains limited. Studies have shown improved patient understanding and outcomes when using 3D models in complex orthopaedic fractures with custom prosthesis and head and neck surgery [2,3,4,5,6,7,8]. In neurosurgery and cardiac surgery, they aid in both training and perioperative planning [9,10,11,12,13,14,15]. However, general abdominal surgery literature remains scarce, with few but increasing references describing their use in soft tissue surgery or communication processes [16]. Notable paediatric applications include thoracoscopic models for diaphragmatic hernia repair [15], pre-operative planning for paediatric tumours [17,18,19], and rare studies assessing communication benefits, mostly in the field of paediatric congenital heart disease [10,11,20]. Very recent reviews specifically address 3D modelling/printing in neonatal surgery [21] and emphasise the specifics of the topic by providing a very good overview of the current emerging practices [22,23,24].

Principally, extra-cerebral soft tissue segmentation in paediatric surgery presents unique challenges due to the complex anatomy of rare diseases and the need for MRI imaging to avoid radiation and provide high-contrast images. To date, 3D modelling often requires a labour-intensive process of manual segmentation [25], as deep learning algorithms are still in the early stages of development, using mostly research pipelines and in-house methods which are not yet usable in clinical practice [26,27,28]. The inclusion of tractography mapping for peripheral nerves could further enhance model precision and surgical safety, especially in tumour and malformation surgeries [25,29,30,31,32,33].

The primary aim was to assess the impact of 3D patient-specific models, enhanced with tractography, on physician–parent communication. The secondary aims were to assess its impact on surgical decision-making and help establish a streamlined and safe image post-processing workflow within a non-university public hospital setting, facilitating the routine use of these models in clinical practice by physicians without a coding or mathematical background, such as the authors. Through interdisciplinary collaboration between surgeons, radiologists, medical physicists, and a 3D laboratory, this initiative anticipates improved outcomes in paediatric surgery, both in terms of clinical efficacy and patient engagement.

Briefly overviewed, this prospective study evaluated the technical feasibility and clinical impact of patient-specific MRI-based 3D models, including peripheral nerve tractography, in children with rare tumours and malformations. Using questionnaire-based assessments and an optimised post-processing workflow, the study demonstrated that high-quality, manually generated 3D models can improve communication and support clinical decision-making while underscoring the need for clinically approved automated MRI-based segmentation tools.

## 2. Literature Overview

A qualitative comparison of the literature overview is displayed in Table 1. It includes only studies that have common characteristics with the current study regarding the population (children), the imaging method (MRI), or the clinical application (communication and planning). None of them fill all the criteria, and this overview allows for accurately identifying the originality of this work despite its small sample size.

## 3. Materials and Methods

**Study Design**: this single-centre prospective study was conducted between 2021 and 2024 at the Cantonal Hospital of Aarau (KSA), Switzerland. The primary endpoint was the consultation at which the patient-specific 3D model was presented to the patient or their family. A parallel secondary endpoint was the presentation of the 3D model to the referring physician before surgery, when applicable. A third secondary endpoint aimed to define an effective and secure digital workflow for producing patient-specific 3D models.

**Patients**: the study included 21 patients, aged between 3 months and 18 years, referred from the general paediatric clinic, the paediatric surgery department, and the emergency department. We included all paediatric patients within this age range who presented with rare tumours or malformations located in the head, thorax, abdomen, pelvis, or limbs. Patients with cardiac or central nervous system conditions were excluded. An informed consent form was signed by all participants or their parents prior to enrolment in the study. The study was approved by the local ethics committee (EKNZ 2021-01328).

**MRI Protocol**: only pathologies requiring MRI were included in the study. All imaging was performed using a Siemens 3T MRI scanner (Vida, Siemens Healthineers, Erlangen, Germany). No research-specific MRIs were conducted and only the local standard clinical MRI protocols for first diagnosis or follow-up were used. These protocols included an extra isotropic T2-weighted sequence with the following parameters: 0.9 × 0.9 × 0.9 mm^3^ voxel size, TE/TR = 106/1300 ms, flip angle (fa) = 120°, 2 averages, TA = 3:25 min with field of view (FOV) and matrix adapted to the region and age of the patient, to ensure optimal 3D segmentation. When clinically relevant for surgical planning, an additional diffusion-weighted sequence (DWI) was acquired for peripheral nerve tractography (2 mm slice thickness, TE/TR = 79/3400 ms, 2 averages, b800, 30 directions, TA = 6:05 min, with FOV and matrix adapted to patient age and anatomy). Given the variety of pathologies, each case required close collaboration between the principal investigator (COM), radiologists/neuroradiologists, and the MRI supervising physicist (JB).

**Post-Processing**: This is an original five-step post-processing workflow designed by the first author (COM), based on the expertise acquired during her PhD in this field [15].

### 3.1. Organ Segmentation

Once a patient was enrolled, the MRI was scheduled, and the raw data were directly retrieved from the scanner and uploaded into 3D Slicer^®^: https://www.slicer.org [35]. 3D Slicer^®^ is a free, open-source software platform for medical image informatics, image processing, and three-dimensional visualisation. It supports a wide range of applications, including surgical planning, image-guided therapy, and research in medical imaging. The platform is extensible, allowing users to develop custom modules and integrate various medical imaging tools. The 4.10 stable release of 3D Slicer^®^ was used in this study, without any in-house-developed plugins. Using the Segmentations module, all neighbouring anatomical structures were manually segmented (LH, COM) on the isotropic anatomical T2-weighted sequence. A senior radiologist (TO) reviewed all segmentations, corrected them when necessary, and validated the results. The only filtering operation applied was the built-in smoothing option of the module, used to achieve a more aesthetically pleasing final rendering. The 3D models were then automatically generated and visualised in 3D Slicer^®^.

Segmentation also included the proximal region of interest (ROI) of adjacent nerves, for example, the sacral canal when sacral plexus tractography was required. These segmentations, deliberately drawn with generous boundaries to maximise the likelihood of capturing the nerve signal, were performed on the diffusion-weighted imaging (DWI) sequence and subsequently served as ROIs for ROI-based tractography.

### 3.2. Tractography

When clinically appropriate, deterministic ROI-based tractography of neighbouring peripheral nerves was performed (COM) using the tractography tools available in 3D Slicer^®^. Diffusion-weighted imaging (DWI) sequences were imported directly from the MRI scanner rather than from the PACS, as metadata—particularly diffusion gradient directions—may otherwise be compressed or lost. During importation, the data were not anonymised, and patient identifiers were restricted to standard alphanumeric characters to ensure compatibility with post-processing. This step is highly dependent on the MRI system and must be coordinated with the manufacturer’s application engineers; otherwise, the diffusion sequence may not be importable into 3D Slicer^®^.

DWI data were subsequently converted into anonymised diffusion tensor images (DTIs) using the DTIEstim module (Diffusion → Diffusion-Weighted Images → DTIEstim). The resulting DTI datasets were then used for ROI-based tractography with the Tractography ROI Seeding module (Diffusion → Tractography → Tractography ROI Seeding), without the use of label maps. Tractography parameters were individually adjusted according to DTI image quality, diffusion signal intensity, nerve calibre (varying with patient age), and anatomical location. For sacral plexus tractography, the parameters were as follows: Start Threshold 0.1, Minimum Length 10 mm, Maximum Length 800 mm, Stopping Criterion—Fractional Anisotropy with a stopping value of 0.25, Stopping Track Curvature 0.7, Integration Step Length 0.5 mm, and Seeding Label 1 (corresponding to the number of the previously segmented ROI, i.e., the sacral canal in this example).

### 3.3. Exportation

Each anatomical structure was exported in stereolithography (STL) format using the Segmentations module, except for the nerve tracts. These were exported in polygon file format (PLY) using the “Export Tractography to PLY” module (Diffusion → Import and Export → Export Tractography to PLY).

### 3.4. Model Optimisation

Polygon counts were subsequently reduced (limited to approximately 20,000 polygons overall, including all organs) using Meshmixer^®^ [30] to optimise the models and enable smooth visualisation at the end of the post-processing workflow. Meshmixer^®^ is a free 3D modelling software developed by Autodesk for working with triangular meshes. In this study, only the mesh-reduction function was used. Polygon reduction was performed via the following pathway: Select All (STL file of the organ) → Edit → Reduce, and the operation was repeated as necessary to achieve an optimal balance between visual quality and file size within the predefined polygon limit. PLY files corresponding to peripheral nerve tractography were also reduced and subsequently re-exported in STL format, similarly to the other anatomical structures.

### 3.5. Visualisation

The optimised models (STL) were then imported into Vectary^®^ at https://www.vectary.com for final rendering. Vectary^®^ is a web-based 3D design tool that combines 3D modelling, product visualisation, and augmented reality (AR) features. It is user-friendly and ideal for designers, allowing for easy collaboration and real-time editing in the browser. Vectary supports both beginners and professionals with its intuitive interface and customizable 3D templates. In this study, Vectary^®^ was used for further editing of the 3D models, including colour coding, transparency adjustments when relevant, and structural highlighting to better visualise the pathology (malformation or tumour), resulting in an aesthetically refined yet anatomically realistic patient-specific model. Due to motion artefacts between the two MRI acquisitions, no automatic registration between the anatomical T2-weighted and diffusion sequences was feasible; therefore, nerve tracts were manually registered and integrated into the final 3D anatomical model based on the first author’s (COM) anatomical expertise in this field [20,24,25,26,27,28], under the supervision of the senior radiologist (TO).

For one patient, the 3D model was printed including the skin, as an aid for surgical planning with the collaboration of the hospital’s 3D laboratory [36].

**Evaluation Questionnaires**: During the consultation, the pathology was routinely explained to the participants, clinically and through an explanation of the classical 2D black-and-white MRI images on the hospital computer.

Immediately afterwards, an e-mail with 3 links was sent, which all participants opened on their own mobile device (phone).

The first link directed them to the initial part of the evaluation questionnaire, created in Google Forms^®^ and designed based on tools from the existing literature: a 5-point Likert scale, ranging from “Strongly disagree” to “Strongly agree” [10,11]. This section focused on the general understanding of the disease and proposed treatments. After completing the first section, participants were asked to access the second link, again on their mobile device, which led to their personalised 3D model in Vectary^®^. The third link led to the second part of the questionnaire, repeating the initial questions and adding five additional items specifically evaluating the 3D model. Meanwhile, the referring physician completed a similar questionnaire after reviewing the 3D model, to assess its perceived impact on clinical or surgical management.

**Statistics**: all questionnaire responses were electronically collected and analysed using R-software version 4.3.315 [37]. A statistical test was only performed on the pre- and post-3D-model-presentation questionnaires using a paired Wilcoxon’s test, the patient being his/her own control. The two other questionnaires (3D model evaluation by the participant and the referring physician) were only analysed descriptively, a statistical analysis with a control group being not feasible with such a group of rare diseases, even with a larger study group. The internal consistency of the questionnaires was assessed using Cronbach’s alpha, calculated with the psych package in the same R-software version 4.3.315.

**Patient data protection**: this was ensured by the anonymisation of clinical, radiological, and questionnaire data from both patients and physicians in a secure database.

## 4. Results

All 21 patients’ pathologies are displayed in Table 2, Table 3 and Table 4, including 7 girls and 14 boys (ratio 1:2) with a median age of 24 months (min–max: 0–204), along with their anonymous 3D models, which are available online.

All patients completed questionnaires both before (with systematic clinical und classical radiologic information) and after being shown a 3D model of their child’s anatomy.

**Figure 1:**
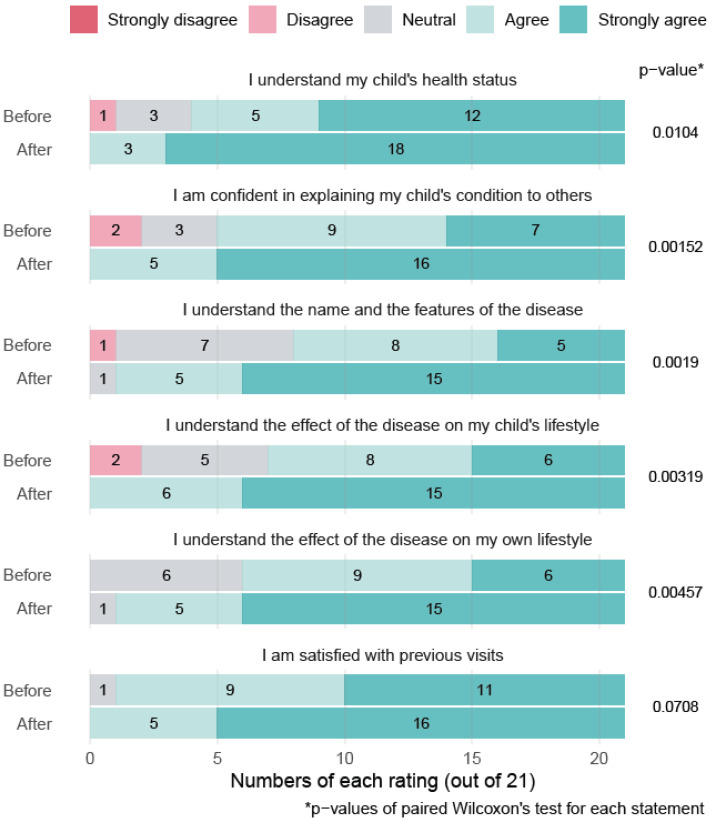
Ratings from patients before and after 3D model presentation.

The results revealed a marked improvement in their comprehension of the disease, confidence in discussing it, and understanding of its impact on daily life. Statistically significant gains were observed across all evaluated areas, except for general satisfaction with previous visits, which was already high and showed no significant change (Figure 1). The first part of the questionnaire about the general understanding of the child’s pathology demonstrated good internal consistency (Cronbach’s alpha = 0.81; 6 items, *n* = 11). The second part of the questionnaire also showed good internal consistency (Cronbach’s alpha = 0.86; 11 items, *n* = 11).

**Figure 2:**
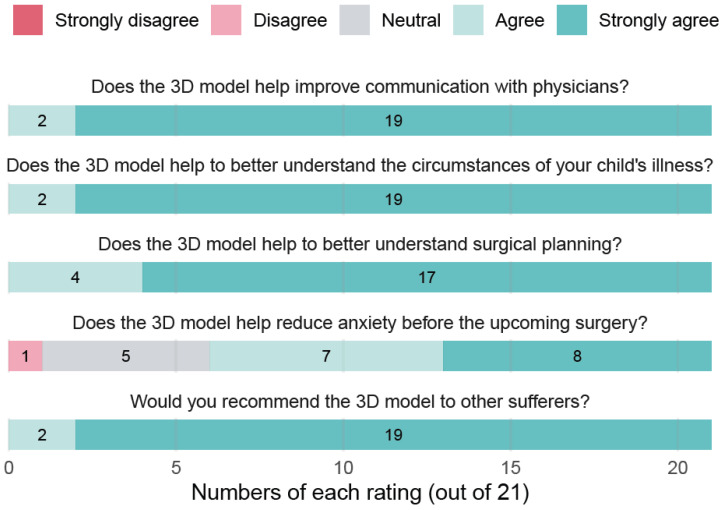
Ratings from patients after 3D model presentation.

Following the 3D model presentation, nearly all patients expressed strong agreement that the models improved their communication with physicians, clarified their understanding of the illness and surgical planning, and were worth recommending to others. A large majority also felt that the models helped reduce their anxiety before surgery (Figure 2). Despite assessing related but not identical dimensions, the third questionnaire focusing only on the perceived usefulness of the 3D model demonstrated very good internal consistency (Cronbach’s alpha = 0.88).

**Figure 3:**
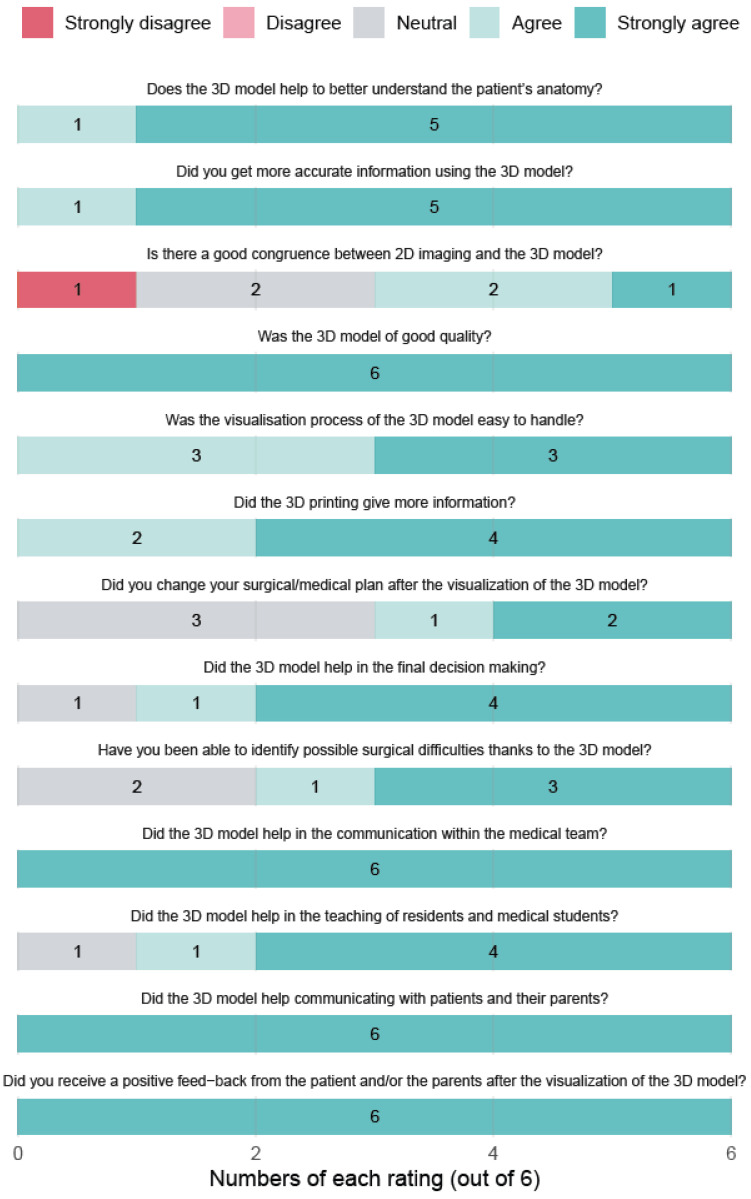
Ratings from physicians after 3D model presentation.

Physicians’ feedback was equally positive. Six doctors involved in the study consistently rated the models as extremely useful for improving communication, enhancing surgical planning, and supporting education. The overall impression among professionals was that patient-specific 3D models represent a valuable addition to clinical practice, particularly in complex paediatric cases (Figure 3). In one polymalformative young patient (#13, 6 months old), the 3D model supported the surgical decision to perform both a distal bowel pull-through and excision of a meningocele during a single operation, thereby avoiding an additional round of general anaesthesia without increasing the risk of infection. Notably, the skin incision required for the anoplasty was located approximately 2 cm away from the incision needed for meningocele excision, resulting in an intact skin bridge that was only identifiable preoperatively on the 3D model. This visualisation facilitated discussion with the parents and supported decision-making for a combined digestive (non-sterile field) and neurosurgical (sterile field) procedure while minimising infectious risk.

Despite the small sample size, the clinician questionnaire demonstrated good internal consistency (Cronbach’s alpha = 0.84, *n* = 6).

## 5. Discussion

These preliminary study results seem to support the role of 3D modelling in improving patient and physician experiences, particularly by enhancing mutual understanding and emotional preparedness in the context of rare paediatric diseases. It also brought up relevant information for physicians in planning the surgery of some complex cases.

Three-dimensional imaging is increasingly being integrated into daily clinical practice, helping to democratise anatomical understanding for parents and children and improving the comprehension of their pathology. However, the 3D reconstruction process from MRI images remains extremely time-consuming (5 to 8 h depending on the complexity of the anatomy). In this study, the choice of manual segmentation was supported by the first author’s experience (COM) in that field. The segmentation time with the available semi-automated segmentation tools and the numerous manual corrections always required in those rare paediatric pathologies is equal to the 100% manual segmentation time and is often less anatomically precise. There is a lack of software capable of automatically performing this task from MRI data efficiently, in contrast to CT scans, which rely on greyscale differentiation in Hounsfield units.

MRI image interpretation and 3D reconstruction require advanced anatomical knowledge. The future lies in the development of software based on deep neural networks, trained on large datasets of images manually annotated by experts in anatomy. Those pipelines are yet to be integrated in clinical practice [38,39,40,41,42]. The challenge of building such large datasets is even greater in paediatric populations, due to very a very wide anatomical variability of malformations, growing organs, and the rarity of diseases. This prospective study used a specific T2 isotropic sequence required for 3D modelling that is not yet included in the standard MRI protocols of most hospitals. There is, to date, no larger external database of MRI isotropic sequences on paediatric pathologies available. Because it is not yet routinely performed, it prevents any retrospective use of adequate MRI data. A comparison with automatic or semi-automated segmentation would indeed be very interesting. There are firms that have recently begun to offer such methods, but the costs are prohibitive and not yet reimbursed by classical health insurance: the yearly fee of Visible Patient^®^, for example, providing very qualitative 3D models based on MRI including paediatric pathologies, is 10,000 euros. The main reason for this is the lack of complete automation in MRI segmentation.

A distinctive feature of the 3D models of this study is the inclusion of the 3D model of peripheral nerve tractography in cases where this added value to the patients’ management. For instance, in the case of a supracondylar fracture (Patient 20), conventional MRI image analysis had suggested a nerve interruption, which was contradicted by the tractography results: the median nerve appeared intact and continuous. This helped in avoiding a high-risk reoperation, and the patient was successfully treated with ergotherapy for her postoperative motor deficit and paraesthesia. A comparison of the ROI-based deterministic tractography method used in this study with more advanced tractography probabilistic methods would have been interesting. However, mastering those very complex post-processing steps is not realistic for a physician without a coding or engineering background.

One intermediate solution, while awaiting the development of automated 3D reconstruction software from MRI usable in routine practice, would be to have these reconstructions reimbursed by the health insurance system, of course using EU- and/or FDA-approved software. Additionally, dedicated time should be allocated to practitioners with the necessary anatomical expertise.

From the perspective of statistical data analysis, each patient served as their own control, allowing for statistically significant results. Given the pilot nature of the study and the small sample size, the internal consistency of the questionnaires was considered acceptable. Unfortunately, it was not possible to establish a control group for the questionnaire analysis involving physicians due to the wide heterogeneity and rarity of the pathologies involved—a characteristic particular to paediatrics. Furthermore, for ethical reasons, it is difficult to gather MRI data from a control group of healthy children undergoing MRI scans.

Overall, all patients responded quite positively to the 3D models. It is noteworthy that only one case (Patient 11 with a nephroblastoma) provoked increased anxiety in a parent, likely due to the malignant nature of the diagnosis and the inherently sensitive psychological context. Despite the small effect size, a tangible positive effect on patient–physician communication could be delineated, which needs to be confirmed by other, still rare, studies focusing on communication using 3D imaging in paediatric surgery [43].

This study also enabled the establishment of an effective interdisciplinary collaboration between paediatric surgeons, radiologists, and the hospital’s 3D laboratory. In only one case, the 3D model of a patient with a complex high anorectal malformation associated with a dysraphism was 3D-printed. This 3D object included the printing of the skin from the lower back to the bottom and had a tangible impact on surgical planning: it enabled the surgical decision to perform both the pull-through of the distal bowel and the excision of the meningocele (patient 13) during the same operation and avoided an additional round of general anaesthesia without increasing infectious risk. Most of the recent literature in paediatric surgery focuses on 3D printing [21,22,23,24,34]. However, the digital 3D model alone may be equally or more effective, faster, and more economical. It already provides sufficient and adequate information to enhance communication and planning in most of the cases [21]. Three-dimensional printing should only be performed when providing additional information, as in the example above.

The study also led to the development of an efficient workflow using multiple 3D visualisation software solutions that are free and available on the market. The lack of individuals possessing mathematical, coding, and advanced anatomical knowledge altogether is one of the main obstacles to making real progress in this field. Paediatric surgeons with the anatomical knowledge but no coding or engineering background need user-friendly interfaces, with minimal post-processing steps. This preliminary study aimed at defining an accessible workflow for a physician, in a non-university hospital, without access to any collaboration with researchers in the field. No specific segmentation operations were used (everything was performed manually), except for the smoothing option to upgrade the aesthetic result. No image enhancement, such as denoising tools for the DWI images, for example, was used. The relative simplicity of the proposed solution reflects a compromise for improving the reproducibility of 3D modelling and broadening its applications. A comparison with an alternative workflow would have been interesting but would have borne inherent biases and therefore not have improved the quality of the study, which is mainly a proof of concept with limited ambition. Nonetheless, this approach will continue to be offered to selected patients with complex anatomies, in collaboration with other specialties such as orthopaedics, urology, and oncology, both within this hospital and in future partnerships with other hospitals of the region.

## 6. Conclusions

This preliminary study demonstrates that patient-specific 3D models may significantly enhance communication between paediatric surgeons, patients, and their families. In the context of rare and complex paediatric diseases, these models can offer a tangible, comprehensible representation of pathology that goes beyond the limitations of traditional 2D imaging. These findings showed an improvement in patient and parent understanding, confidence, and emotional comfort after the use of 3D models, with physicians also recognising their value in planning and interdisciplinary discussions. This preliminary study is well positioned within the current literature, highlighting the importance of establishing a safe and efficient image post-processing workflow in public hospitals to enable routine implementation. Personalised 3D models, especially when including tractography, have the potential to transform paediatric surgical care, not only as a planning aid but also as a powerful communication and educational tool. The medical community should support the development of MRI-based automated segmentation software using deep neural networks that are clinically approved, affordable and/or reimbursed, and usable in routine practice.

## Figures and Tables

**Table 1:** Literature overview.

Author (Year)	Study Design (*n*)	Population	Imaging + 3D Method	Software	Clinical Use	Findings
Schenk et al. (2004) [18]	Prospective (*7*)	Paediatric tumours	MRI + Semi-automated + Manual	VG Studio Max Commercial Off-line	Planning	Helpful
Günther et al. (2006) [19]	Prospective (*NA*)	Paediatric tumours	MRI + Semi-automated + Manual	VG Studio Max Commercial Off-line	Planning	Helpful
Weinstock et al. (2015) [15]	Prospective (*4*)	Paediatric neurovascular malformations	MRI + CT + Semi-automated	In-house	Planning	Helpful
Biglino et al. (2017) [10]	Prospective (*20*)	Congenital heart disease in adolescents	MRI + Semi-automated	Simpleware Ltd. Commercial, Off-line	Communication	Helpful
Francoisse et al. (2021) [23]	Review	Paediatric patients	All	All	All	Helpful
Anand et al. (2022) [24]	Review	Paediatric patients	All	All	All	Helpful
Yang et al. (2024) [34]	Review	Paediatric patients	CT+ Automated	Not relevant	Communication	Helpful
Grefen et al. (2025) [20]	Prospective (*57*)	Congenital heart disease	CT+ Automated	Mimics 24.0	Communication	Helpful
Girón-Vallejo et al. (2025) [21]	Prospective (*8*) + Review	Neonatal surgery	MRI + Semi-automated + Manual	3D Slicer + Simpleware	Communication Planning	Helpful
Muller et al. (this work)	Prospective (*21*)	Paediatric tumours and malformations	MRI (T2+DWI) + Tractography + Manual	Slicer + Meshmixer + Vectary Free + Online	Communication Planning	Helpful

**Table 2:** Patients with tumours.

Patient	Age (Months)	Sex (M/F)	Tumour Type	3D Model in Vectary
#1	10	F	Veinous Lymphatic Malformation left parotidal gland	https://app.vectary.com/p/6bVocKzx5tqnAC5ajt0U3Y (accessed on 13 January 2026)
#2	21	M	Dermoid Cyst of the nose	https://app.vectary.com/p/6ArIudvuNgiPHTi9gTkSzR (accessed on 13 January 2026)
#3	57	M	Venous Malformation Knee left	https://app.vectary.com/p/72ykG4sKjEpbaqFTm5rDZa (accessed on 13 January 2026)
#4	137	M	Schwannoma axillar left	https://app.vectary.com/p/1VrMMlPBJB1tT969dn0cA6 (accessed on 13 January 2026)
#5	168	M	Testicular asymmetry links	https://app.vectary.com/p/5kGFVKmcIWQJYC6KHOfgrh (accessed on 13 January 2026)
#6	8	M	Atretic occipital Cephalocele	https://app.vectary.com/p/3C6xg6xhOmtTHdmh0f7ily (accessed on 13 January 2026)
#7	14	M	Dermoid Cyst right Orbit	https://app.vectary.com/p/5amgKAQbtk2rqPjIc27MXF (accessed on 13 January 2026)
#8	9	M	Peri-anal Lipoma	https://app.vectary.com/p/4TdzS7tcSKGRSTIeex9SF9 (accessed on 13 January 2026)
#9	0.3	F	Sacrococcygeal Teratoma	https://app.vectary.com/p/0geo5QdvKoZygTYNEBA6EN (accessed on 13 January 2026)
#10	8	F	Neuroblastoma right	https://app.vectary.com/p/5d17X6oa4nerTKowz2UNMh (accessed on 13 January 2026)
#11	56	M	Nephroblastoma left	https://app.vectary.com/p/61BPwaekTq3sBeMIDsPHlI (accessed on 13 January 2026)

**Table 3:** Patients with malformations.

Patient	Age (Months)	Sex (M/F)	Malformation Type	3D Model in Vectary
#12	178	F	High anorectal Malformation with T21	https://app.vectary.com/p/0yQtPwg4AKht0V5qBDjkak (accessed on 13 January 2026)
#13	6	M	High anorectal Malformation with Currarino Syndrome	https://app.vectary.com/p/0nDxMftc9TsZRI8sBqTEU1 (accessed on 13 January 2026)
#14	84	M	Rectal Duplication	https://app.vectary.com/p/7cJ4zs9g8csPPgXeFD2P2l (accessed on 13 January 2026)
#15	3	F	Tethered Cord	https://app.vectary.com/p/0XKYcFRQT1OzAfCFXw3LyP (accessed on 13 January 2026)
#16	108	M	Rectal Stenosis with Currarino Syndrome	https://app.vectary.com/p/1y3bMQZ8UggDgYFI2SfntP (accessed on 13 January 2026)
#17	204	F	Caudal regression syndrome	https://app.vectary.com/p/6W3WAYRTPh95laKPKOxPCe (accessed on 13 January 2026)
#18	24	M	Rectal Duplication	https://app.vectary.com/p/1OQKCrLu3avQsJeFQqV5kV (accessed on 13 January 2026)
#19	12	M	Seminal vesicle Cyst left	https://app.vectary.com/p/3WLEBHAJxtzTBGTob4IROx (accessed on 13 January 2026)

**Table 4:** Miscellaneous.

Patient	Age (Months)	Sex (M/F)	Miscellaneous	3D Model in Vectary
#20	84	F	Supracondylar Fracture Type IV left	https://app.vectary.com/p/78RtNeQyr5WxwS6uIBoRKN (accessed on 13 January 2026)
#21	84	M	Pancreatic Pseudo-Cyst	https://app.vectary.com/p/4vZtzrG2j9Jsi3bmQ9mGq0 (accessed on 13 January 2026)

## Data Availability

The data presented in this study are available on request from the corresponding author due to privacy restrictions.

## Abbreviations

The following abbreviations are used in this manuscript:

3D	3-dimensional
MRI	magnetic resonance imaging
CT	computed tomography
DWI	diffusion-weighted images
DTI	diffusion tensor images
ROI	region of interest
STL	stereolithography
PLY	polygon file format
